# Can All Behavioral Problems Be Blamed on Equine Gastric Ulcer Syndrome?

**DOI:** 10.3390/ani15030306

**Published:** 2025-01-22

**Authors:** Ben Sykes, Amy Lovett

**Affiliations:** 1 BW Sykes Consultancy, Coffs Harbour, NSW 2450, Australia; 2 School of Agricultural, Environmental and Veterinary Sciences, Charles Sturt University, Wagga Wagga, NSW 2650, Australia; alovett@csu.edu.au

## 1. Introduction

Behavioral problems are a common complaint in equine practice, particularly in sport horses. This review aims to discuss ‘behavioral problems’ as a possible manifestation of pain, with specific reference to equine gastric ulcer syndrome (EGUS). The article includes a discussion of the range of differential diagnoses that warrant consideration and the authors’ clinical approach to the diagnostic evaluation of pain-based behavior as a problem presented to equine clinicians.

## 2. Is All Undesirable Behavior “Bad Behavior”?

Recently, there has been momentum in the literature encouraging equine professionals to reassess the notion of horses being behaviorally “good” or “bad”, instead encouraging the consideration of underlying diseases, particularly painful diseases, as a potential, or even likely, cause of behavioral problems [1]. The nomenclature in this field is poorly defined, but for the purpose of this review, undesirable behavior is defined as any “behavior that exceeds the boundaries of acceptable behavior” in the context of a trained horse. Aversive behavior is specifically defined as “undesirable behavior in response to an applied non-noxious stimulus”, e.g., unwanted behavior demonstrated in response to applying tack, girthing, or in response to the appropriate use of a bit or a rider’s cues when ridden.

When considering the potential presence of an underlying painful disease, it is important to establish that the intention of the definitions provided above is to acknowledge that not all undesirable behavior is aversive, and as such not all undesirable behavior can be attributed to pain, e.g., while often undesirable, most stallion or mare estrus behavior is behaviorally normal and not in response to an aversive stimulus. It is equally important to recognize that horses have individual personalities, that genetic breed differences exist with anxiousness and excitability (arousal level) varying most between breeds [2,3], and that these factors can influence the interpretation of “normal” across different horse types.

Lastly, not all aversive behavior is pain-based, aversive behavior could also be a manifestation of fear, e.g., a reluctance towards trailer loading or to leaving a social group, and it should be noted that in an untrained horse these would be considered appropriate responses [4]. The development of aversive behavior in a trained horse (a horse that is familiar with its environment and expectations) is particularly important in a veterinary context, because such behavioral changes could be due to a motivation to avoid the agent causing them pain, whether the non-noxious stimulus itself is painful (allodynia) or the horse associates the applied stimulus with imminent pain (e.g., anticipation of pain).

## 3. When Does Pain Become Personality and What Is the Impact of Training?

It is well recognized in humans that pain, particularly chronic pain, can result in a ‘negative emotional state’ (e.g., fear, anxiety, depression, and distress), poor sleep, and deficiencies in coping with changes in one’s environment [5,6]. By extension, it is possible behavioral traits in a horse such as aggression, dullness, fear, anxiety, or distress could be due to underlying pain as opposed to being explained away as a horse’s innate personality of being ‘quiet’, ‘grumpy’, ‘spooky’ or ‘stressy’. This is particularly true when changes towards aversive behavior are observed.

To quantify this, methods for the clinical recognition and objective quantification of a horse’s behavior and pain continue to receive attention in the equine scientific literature. This includes multiple variations of equine pain scales, including the ‘horse grimace scale’ and the ‘ridden horse pain ethogram’ (RHpE) [7,8,9]. The potential confounding roles of training, rider/handler skill level, breed, arousal level, and personality are particularly important to consider when applying pain and behavioral assessment tools. In the context of the RHpE, it is essential to acknowledge that it has been primarily validated in a specific population of well-trained sport horses ridden by experienced riders. Training and rider skill play a significant role in rideability assessment outcomes in the RHpE, such as head position, gait, bit behavior, and interaction with the environment [8]. Thus, the authors caution against the unreserved application of RHpE to all horse populations and recommend considering factors such as breed, rider ability, and the horse’s previous training when interpreting its results. It is also important to note that because the RHpE consists of a constellation of clinical signs, whereby a total cumulative behavior score of ≥8 (out of 24) likely indicates the presence of pain, over interpretation of any one element in isolation should be avoided.

Observed behavior could be a direct pain response to the immediate stimulus (placing a saddle and girth tightening) or aversive behavior that persists beyond the resolution of any apparent cause of pain, suggesting that a learned, anticipatory response may be present. It is not surprising that horses can make anticipatory associations, and it has recently been demonstrated that horses are indeed capable of “planning” [10]; however, the integration of this knowledge into the management of chronic pain-based diseases (e.g., lameness, EGUS) is yet to be widely adopted in clinical practice. Instead, the outcome of clinical decision-making is still commonly disease-based, e.g., a goal such as endoscopic EGUS or lameness resolution during a follow-up examination.

Clinically, if a pain-based disease is resolved at a recheck appointment, but the horse is still affected behaviorally, it begs the following questions:

- From the horse’s perspective, has the problem really been solved?
- Was the original diagnosis incorrect or incomplete?
- Is there a learned behavioral element that needs to be addressed in parallel with treating the primary disease(s)?

## 4. EGUS and Aversive Behavior

Anecdotally, aversive ground behavior is commonly attributed to EGUS in clinical practice and collective clinical experience suggests that utilizing gastroprotectant therapy in such horses commonly improves the aversive behavior presented. Within the scientific literature, the association between changes in behavior and EGUS is observed in some studies [11,12,13], yet not in others [14,15,16]. In a population of horses diagnosed with equine squamous gastric disease (ESGD), 25% presented with girthiness [12], and in a population of horses with equine glandular gastric disease (EGGD), 31% presented with girthiness [13]. In the same studies, 32% of horses with ESGD and 33% of horses EGGD presented with a history of ‘changes in behavior’ [12,13]. In an earlier study in a show horse population, horses with a ‘nervous’ disposition, based on how a horse responded to training and new stimuli, were more likely to have ESGD when compared to horses with a ‘quiet’ or ‘normal’ demeanor [11]. Furthermore, the relationship between ESGD and changes in eating behavior is well documented [12,17,18].

On the contrary, an attempt to correlate commonly ascribed undesirable ground behaviors to EGGD found no relationship between the presence of disease and owner-reported clinical signs [15]. Similarly, little or no relationship between the resolution of EGGD lesions gastroscopically and the resolution of owner-reported clinical signs has been reported in the literature [13,19]. These findings have led some commentators to draw into question the relevance of EGUS as a differential diagnosis for the clinical presentation of aversive behavior (Sue Dyson, personal communication). Supporting this, in stark contrast to the large body of evidence demonstrating a relationship between aversive behavior and musculoskeletal disease, until recently there has been little evidence in the peer-reviewed literature evaluating specific aversive behavior traits and EGUS in isolation.

Some specific presentations have been investigated, and when girthiness was evaluated as an isolated problem at presentation to a university referral hospital, gastroscopy was performed in 13 of the 37 studied horses, of which 12/13 (92%) were diagnosed with EGUS [20]. Further, all of the horses showed behavioral improvement with omeprazole treatment and 10/12 had complete resolution of girthiness [20]. Although this was a small population of horses in a descriptive retrospective study, the reported clinical improvement with treatment is supportive of EGUS as a possible cause of girthiness. Unfortunately, the study did not include descriptions of whether the 12 horses diagnosed with EGUS had ESGD, EGGD, or both. In the only study to date that applies the RHpE to horses diagnosed with EGUS, strong positive correlations between ESGD and EGGD and the RHpE score were identified [21]. This study provides the first direct evidence of a relationship between changes in aversive ridden behavior and EGUS. Although the horses underwent a lameness exam during the 12-week study period, the results of the lameness exams were not described, but as gastric lesions improved over time so did RHpE scores [21], further supporting a potential causative association. Although they provide only a small body of evidence, the findings of these studies support the collective anecdotal clinical experience of associating EGUS with aversive behavior, during both tacking and riding, and supports the inclusion of EGUS as a key differential when evaluating aversive behavior.

## 5. Differential Diagnoses for Pain-Based Aversive Behavior

Musculoskeletal disease, including poor saddle fit, is a clear cause of aversive behavior [8,22,23], and in the authors’ opinion, it should be considered the number one differential if a horse’s sole presenting complaint is aversive behavior during riding or tacking up. The second key differential for pain-based aversive behavior, in the authors’ opinion, is EGUS. The relative likelihood of EGUS as a differential is heavily influenced by either a history or the clinical presence of gastrointestinal disease, e.g., a history of colic within the last 3–6 months. Thus, when the horse’s history or clinical presentation suggests gastrointestinal disease, alongside aversive behavior as a presenting problem, EGUS should be the primary differential. Furthermore, if the horse’s history additionally includes changes in eating behavior or unexplained weight loss, ESGD should be considered specifically.

Like EGUS, dental disease is anecdotally associated with ridden aversive behavior (e.g., bit aversion), yet little evidence exists in the peer-reviewed literature to support this position. This warrants a consideration of whether this represents a true dissociation between the presence of disease and aversive behavior, or if the lack of evidence is due to other factors such as inadequate or inappropriately designed studies. In support of the relationship between dental disease and aversive behavior, recent studies have described a relationship between complex dental pathology and behavioral changes (including aversive behavior towards bridling and bit use, as well as changes in eating behavior) [24,25]. However, the relationship between simple dental pathology (i.e., sharp enamel points) and behavioral changes is yet to be established in the literature. The absence of proof does not necessarily correlate with a lack of association, especially given the paucity of appropriately designed studies in this area. Thus, the authors weigh the clinical relevance of dental disease as a potential differential for aversive behavior. The prioritization of dental disease as a likely differential should be increased if a horse with aversive behavior also has other coinciding factors in its history, particularly abnormal eating behavior (e.g., dropping feed, quidding) or abnormalities of the head (e.g., facial asymmetry, food packing, halitosis) that are suggestive of more severe dental pathology.

Other differentials for alimentary disease as a cause of pain-based aversive behavior include sand enteropathy and inflammatory bowel disease. One study evaluating EGGD risk factors concluded that “the presence of sand appeared to have a protective effect against EGGD” [26], yet no biologically plausible mechanism was provided. The authors of the present review consider that a protective biological effect is unlikely. Instead, they propose that an alternative interpretation of this observation is that both EGGD and sand enteropathy are common diseases affecting horses within the study population’s region (Finland), that both diseases can have a similar clinical presentation, and, when evaluated in the aforementioned study, horses were either diagnosed with EGGD or sand enteropathy, creating the misconception of a “protective effect”.

The potential for behavioral complaints to be part of the primary presentation of sand enteropathy is supported by a series of studies on the presentation, diagnosis, and management of sand enteropathy [27,28]. Similarly, the authors’ experience is that inflammatory bowel disease can be present alongside aversive behavior as a component of the primary presentation [29]. In considering sand enteropathy and inflammatory bowel disease as differentials, the authors rank them as secondary differentials unless historical or clinical presentation factors suggest otherwise, e.g., significant weight loss despite a good appetite and appropriate nutrition, or changes in fecal consistency. Alongside sand enteropathy and inflammatory bowel disease, sources of pain involving other body systems (urogenital, hepatobiliary) should also be considered when the initial diagnostic approach fails to resolve the primary presentation, or the history or clinical examination suggest that other body systems might be involved, e.g., jaundice suggestive of hepatic disease.

## 6. Comprehensive Approach to Diagnostic Evaluation of Aversive Behavior

The authors’ approach to the diagnostic evaluation of pain-based aversive behavior primarily focuses on the three key differentials described above, with other differentials subsequently considered when appropriate. In order of likelihood, these three key differentials are as follows:

1. Musculoskeletal disease (including saddle fit);
2. EGUS;
3. Dental disease.

The authors’ approach includes performing a basic musculoskeletal and lameness exam, primarily consisting of trotting the horse in a straight line on a firm surface and a visual examination of the limbs and foot conformation, noting any abnormalities for future consideration and further evaluation. The musculoskeletal examination includes palpation of the back and recommendations for evaluating saddle fit if any concerns are present. Gastroscopy should then be performed under standing sedation. Lastly, an oral speculum can be utilized following gastroscopy to facilitate a thorough dental examination while the horse is still sedated. In the authors’ experience, routine blood work (i.e., complete blood count and biochemistry) is unrewarding in the initial stages unless indicated by specific problems identified during history taking or clinical examination. However, it is important to acknowledge that this approach likely varies from population to population, e.g., the likelihood of muscle disease as a differential is influenced by signalment, nutritional, and historical factors and, in some populations, it should be considered a primary differential.

## 7. Bias in Case Interpretation

An individual clinician’s training might heavily influence the initial approach to the clinical presentation of pain-based aversive behavior. Although poorly described in veterinary medicine, specialist bias, which includes a tendency for specialists to recommend treatment solutions for diseases they are most knowledgeable on and capable of delivering treatment for, is well recognized in human medicine, e.g., surgeons are more likely to recommend surgery than non-surgeons [30]. Surgeons and sports medicine-trained clinicians might naturally default to musculoskeletal disease, internal medicine-trained clinicians to gastrointestinal disease, and clinicians with a strong interest in dentistry to dental disease.

Likewise, cognitive biases, such as anchoring bias or confirmation bias, are common in veterinary clinical decision-making [31]. Anchoring bias occurs when a clinician fixates on an initial hypothesis (diagnosis) and fails to investigate other possible hypotheses (diagnoses). Confirmation bias is when a clinician seeks evidence to support a specific hypothesis (diagnosis) and ignores clinical data that might disconfirm the hypothesis (diagnosis). Owners might also be susceptible to confirmation bias based on preconceived opinions that have been shaped by marketing, e.g., an owner who thinks their horse has gastric ulcers therefore presents their horse for gastroscopy, rather than presenting their horse for the actual behavioral complaint. This, in turn, can inadvertently influence the clinician’s approach to the case through selection of a clinician with a particular interest in EGUS, or by inducing an anchoring bias in the attending clinician through the described presentation, i.e., booking the horse for gastroscopy rather than for an evaluation of their aversive behavior.

Given that musculoskeletal disease, EGUS, and dental disease are all common in equids, as discussed below, they potentially become a self-fulfilling prophecy due to the presence of an anchoring or confirmation bias, such that each type of clinician finds an apparent “cause” within their preferred clinical field without acknowledging that there might be an alternative cause or multifactorial contributors to the net outcome of a painful horse, e.g., when a disease in one body system is identified the diagnostic investigation is generally not continued, meaning that other possible contributing diseases might be overlooked.

## 8. Aversive Behavior as a Complex Presentation

In contrast to a disease-based approach, equine practitioners should approach presentations of possible pain-based aversive behavior with a ‘dragonfly-eye’ mind-set (“see through multiple lenses”) where all clinical information is synthesized to provide an increased chance of holistically understanding and subsequently treating the identified underlying pain-based disease(s) [32]. The authors consider the presentation of pain-based aversive behavior to be analogous to the clinical presentation of poor performance [33]. Individual clinicians might default to specific body systems or disease conditions based on experience, but it has also been documented that concurrent multi-system disease within an individual horse is common, e.g., 84% of 275 racehorses presenting for poor performance had more than one potential cause identified when they were subjected to a comprehensive, multi-system evaluation [33].

This information has shaped the authors’ attitude toward the multisystem approach to pain-based aversive behavior described above. Alongside gastroscopy, the addition of lameness and dental examinations adds only a small amount of time to the clinical evaluation of the horse, but they add a depth of valuable information for interpretation of a primary problem that is possibly pain-based aversive behavior. This additional information becomes particularly useful when the response to treatment of the “primary disease”, as measured by behavioral change, is sub-optimal, allowing the treatment plan to quickly pivot to an alternative differential diagnosis. It also allows for the identification of common problems such as poor farriery, poor saddle fit, and dental disease, all of which are relevant to the holistic management of the sport horse as an athlete.

## 9. Assigning Clinical Significance

Assigning clinical significance to the findings of the diagnostic approach described above can be challenging, particularly because disease in all three body systems is common. The prevalence of ESGD in sport horses is commonly reported as 50–70% and a prevalence of EGGD of up to 75% has also been reported in sport horses [34,35]. Likewise, only 32% of low-level one-day event horses were reported to be sound in a recent study [36]. The prevalence of simple dental disease in performance horses is poorly reported, reflecting an inherent bias in the literature away from studying first-opinion dental practice, but anecdotally the prevalence is high.

In the authors’ experience within their clinical population, it is common to identify multiple diseases when a multisystem approach is adopted. This, in turn, shapes the treatment recommendations towards the concurrent management of multiple diseases/body systems rather than focusing on a singular disease to which causation has, rightly or wrongly, been attributed. Considering the above, the authors strongly caution against the clinical fallacy of correlation = causation, which is biased by a clinician’s default approach to aversive behavior by focusing on just one particular body system.

Identifying multiple concurrent diseases further compounds the challenge of assigning clinical significance to any one body system. At one level, identifying a specific etiologic cause is academically rewarding for the clinician, but at another level, resolution of the primary presenting complaint (aversive behavior) is usually more important to the owner and, arguably, the horse. The relative importance of, and the owner’s preference towards, knowing the cause(s) and resolving the problem impacts the authors’ approach to case management.

In the context of this review, when EGUS is diagnosed, the authors prefer to treat the horse with gastroprotectants (omeprazole PO SID for ESGD; omeprazole PO SID and sucralfate PO BID/TID for EGGD or if both ESGD and EGGD are diagnosed) for at least 7 days and have the owners closely monitor the response to such treatment. When aversive behavior is associated with riding (either during tacking up and/or when ridden), it is important that the owner continues to ride the horse during the treatment period so they can assess for possible improvement. When a positive response is seen, i.e., improvement in the reported aversive behavior, treatment is continued as per standard treatment recommendations [34]. When no or a limited response is seen, the relative significance of other findings, particularly mild musculoskeletal disease, is considered. A therapeutic trial with phenylbutazone at label doses for at least 5–7 days is typically recommended in such cases. Although not all musculoskeletal disease responds to this approach, the authors have found it provides useful information and allows them to focus on ongoing evaluation and treatment of the musculoskeletal or gastrointestinal system (or both), and it accelerates the consideration of other differentials further down the diagnostic list. Any dental disease identified during the examination is treated as per standard recommendations as part of the overall health plan for the horse.

Importantly, the above is not a dichotomous “one-or-the-other” approach. In some cases, a singular, likely causative disease is identified based on the patient’s clinical response to treatment, and any other abnormalities are considered clinically insignificant. In other cases, multiple concurrent disease processes are likely relevant, and the clinical presentation of aversive behavior is considered a manifestation of cumulative effects as several small contributors to pain accumulate to exceed the horse’s pain and behavioral thresholds.

## 10. The Role of Learned Behavior

It is also important to consider the potential impact of learned behavior when assessing responses to the therapeutic trials described. A clear response to treatment with resolution of the aversive behavior strongly supports the clinical relevance of the disease that was targeted with the treatment, e.g., when 21 lame horses were assessed via the RHpE before and after the abolition of their lameness with diagnostic analgesia, there was a highly significant decrease in their overall RHpE behavior score as well as a highly significant decrease across the various individual behavioral traits within the RHpE (head position, tail position, facial expressions, and gait) [23]. An additional example (as discussed earlier) was when gastroscopy was indicated for the evaluation of ‘girthiness’, 12/13 horses were diagnosed with EGUS gastroscopically and all 12 horses’ girthiness improved following gastroprotectant therapy [20].

The assessment of behavioral non-responders is challenging, as a failure to respond can be confounded by the impact of learned behavior and it begs the question of whether the diagnosis is being wrongly ascribed as causative or if the diagnosis is, at least partially, correct but the persistence of aversive behavior is a manifestation of a learned response. This is particularly true for anticipatory, pain-based behavior such as girthiness due to a long-standing chronic disease state (such as EGUS), where learned behavior is more likely to be ingrained. As such, the authors consider even a partial response to treatment consistent with the diagnosed disease being at least partly contributory to the horse’s behavioral presentation.

## 11. Managing the Learned Behavioral Response

When a learned behavioral response is suspected, specific behavioral modification is employed. Situations where this is considered appropriate are when the behavior is possibly anticipatory (e.g., girthiness) or long-standing, or when the behavior only partially responded to treatment as described above. Specific behavioral modification focuses on reshaping the relationship the horse has formed with the activity (the applied stimuli, e.g., tacking, or ridden cues). When the behavior is largely anticipatory, modification is focused on the tacking-up period. Pre-exercise feeding, a valuable strategy for reducing ESGD risk [37], forms strong positive associations with the activity, particularly for food-orientated horses, and is a simple and low-cost strategy to employ. Other strategies such as massage and playing specific types of music have been demonstrated to reduce physiological stress responses [38], and can be readily employed ‘tools’ for creating positive associations during tacking.

Reshaping learned behavior that occurs during riding is more difficult. The authors’ approach to this is to encourage owners to limit the number of riders, at least during the retraining period, and to encourage riders to be more accommodating and less punitive, particularly while the horse is reforming its association with a particular activity. Clinically, this is referred to as asking the rider to reset and consider the horse as a “green” horse who is new to learning rather than an experienced well-trained horse that is “expected to already know”. There is no universal training technique for managing a learned riding behavioral response once a primary disease has been resolved with targeted therapy, and a detailed discussion of this is considered beyond the scope of the present review.

## 12. Defining a Successful Outcome

Closing the loop between the clinical presentation and the diagnostic evaluation, the definition of a successful outcome must include a resolution of or an improvement in the primary presenting complaint, i.e., aversive behavior, when a treatment is prescribed for a given diagnosis, and not just medical resolution of the diagnosed disease(s). When this occurs, long-term case management includes making specific recommendations to clients for managing all diseases identified as well as behavioral modifications if required.

## 13. Conclusions—Asking the Right Questions

The disconnect between the clinical experience of diagnosing and treating EGUS as a cause of pain-based aversive behavior and the paucity of literature in this area highlights the need for clinicians and researchers to ensure that the right questions are being asked when attempting to address behavioral clinical presentations in practice and in study design. This should include the use of appropriate and, wherever possible, objective endpoints. In the context of the current discussion, the authors highlight the need for a better definition of “healed” in reference to EGUS, particularly EGGD, as well as validation and application of the RHpE to other body systems. Further exploration of the potential of wearable technology to provide objective measurements that relate to equine behavioral and welfare outcomes might also be advantageous.
